# The work-life balance of general practitioners as a predictor of burnout and motivation to stay in the profession

**DOI:** 10.1186/s12875-022-01831-7

**Published:** 2022-08-30

**Authors:** Erik Bodendieck, Franziska U. Jung, Ines Conrad, Steffi G. Riedel-Heller, Felix S. Hussenoeder

**Affiliations:** 1 General Practice, Dresdener Str. 34a, 04808 Wurzen, Germany; 2 Institute of Social Medicine, Occupational Health and Public Health, University of Leipzig, Ph.-Rosenthal-Str. 55, 04103 Leipzig, Germany

**Keywords:** Work-life balance, Burnout, Physician, GP, MBI

## Abstract

**Background:**

Work-life balance (WLB) is associated with a variety of health-related outcomes in the general population. Since General Practitioners (GPs) play a fundamental role in the health system, we wanted to analyze the associations between their WLB and burnout scores as well as motivation to stay in the profession.

**Methods:**

In September 2019, physicians from various specialties answered a comprehensive questionnaire. We analyzed a subsample of 188 GPs that were working full time, 61.7% were female.

**Results:**

Multivariate analysis showed a beneficial association between WLB and all three dimensions of burnout (Emotional Exhaustion, Cynicism, and Professional Efficacy) as well as the motivation to stay in the profession.

**Conclusions:**

Improving GPs WLB could be a way to reduce physician burnout, strengthen the healthcare system, and attract a new generation of talented physicians.

## Background

Research shows that work life balance is positively associated with wellbeing as well as job and life satisfaction, and negatively with anxiety, depression and mental health problems [1–3]. Although research interest in physician work life balance (WLB) had been growing over the last decade [4–6], the connection between WLB and physician burnout is rarely addressed [7, 8]. However, studies with participants working in healthcare, banking, insurance, and logistics [9, 10], and with orthopedic trainees [11] implicate a connection between WLB and burnout. Burnout is characterized by (a) feelings of exhaustion or energy depletion; (b) increased mental distance from one’s job, or job-related feelings of negativism or cynicism; (c) a reduction in professional efficacy [12]. It is often understood as a three-dimensional construct including exhaustion, cynicism and (reduced) professional efficacy [13]. Due to their prominent role in the health system, general practitioners` (GPs) mental health has implications for themselves as well as for their patients. Research shows that burnout is not only connected to lower career satisfaction and increased depressiveness and hopelessness in physicians [14–16], but also to reduced professional work effort, quality of care, and an increased risk of medical errors [17–19]. With the diverse and severe implications of physician burnout, it is important to better understand its connection with WLB. Hence, in our study we want to contribute to the research by analyzing the connection between WLB and burnout as well as the motivation to stay in the profession in a sample of German GPs.

## Materials and methods

### Study design and sample

In September 2019, 1412 physicians working in the Federal State of Saxony, a region in the Eastern part of Germany, filled out a questionnaire on work strain, health, and work satisfaction. Physicians in the original sample came from a variety of different specialties including internal medicine, surgery, neurology, and prevention. The sample was already used to analyze burnout and WLB in specific groups of physicians, i.e., specialists in internal medicine and physicians with a migration background [20, 21]. For our analysis, we utilized a subsample consisting of all GPs working full time. After removing 39 participants due to missing values, the final sample contained 188 GPs. This study was approved by the ethics committee of the university, and participants have given consent for their data to be used in the research.

### Assessment

We assessed a variety of sociodemographic data that comprised age, gender, marital status, place of residence, and children living in the household as well as number of working hours.

### Burnout

We used the German version of the Maslach Burnout lnventory - General Survey (MBI-GS, [13, 22] which consists of the three dimensions Exhaustion, Cynicism, and Professional Efficacy. After Professional Efficacy had been inverted, weighted average scores of all three scales were added and categorized following the approach by Kalimo et al. [23]. While in theory the aggregated burnout score could range from 0 (= never) to 6 (= every day), participants in our sample scored between 0 and 4.

### Work-life balance

We assessed global, subjective WLB with the German Trierer Kurzskala (TKS-WLB, 24). It consists of five statements that can be answered on a Likert-scale from 1 (= absolutely not true) to 6 (= absolutely correct). Typical items are “I am happy with my balance between work and private life” and “It is hard for me to reconcile work and private life”, and higher scores indicate a better WLB. The scale provides good psychometric properties [24].

### Statistical analyses

SPSS Version 25 was used for the statistical analyses. We used multiple linear regressions to analyze the effects of WLB on burnout and motivation to stay in the profession. We controlled for place of residence and children in the household since both variables showed an association in our previous analyses.

## Results

### Descriptive characteristics

Our sample contained 188 individuals of which 61.7% were female. Table 1 shows the general characteristics of the study population.

**Table 1:** General characteristics of the study population

	Study population (*N* = 188)
Age (Mean)	50.4 (9.3)
Gender (female)	116 (61.7%)
Marital Status	
Married	141 (75.0%)
In a relationship	27 (14.4%)
Single	20 (10.6%)
Place of residence	
Rural (<=5000)	33 (17.6%)
Small (<=20,000)	56 (29.8%)
Medium (<=100,000)	43 (22.9%)
Big (< 100,000)	56 (29.8%)
Working hours per week (including overtime)	48.8 (9.0)
Children in household (“yes”)	96 (51.1%)
Burnout^a^	
None	104 (55.3%)
Some symptoms	78 (41.5%)
Burnout	6 (3.2%)
Work-Life Balance	3.8 (1.3)
Motivation to stay in profession	6.0 (1.4)

*Note*. Continuous variables are given as mean (standard deviation); categorical variables are displayed as numbers (percentages)

^a^Burnout scores were computed and categorized following the approach by Kalimo et al. (2003). Scores were categorized as follows: 0 – 1.49: no burnout, 1.50 – 3.49: some symptoms, 3.50 – 6.00: burnout

### Prediction of burnout and motivation to stay in the profession

WLB exhibited a negative effect on Exhaustion, and Cynicism, and a positive effect on Professional Efficacy and Motivation to stay in the Profession. There was a negative effect of children in the household on Exhaustion and of residing in a rural place on Cynicism, while female gender exhibited a positive effect on Professional Efficacy. Results of the regression analysis can be found in Table 2.

**Table 2:** Prediction of burnout and motivation to stay in the profession by work-life balance (*N* = 188)

	Emotional Exhaustion	Cynicism	Professional Efficacy	Burnout (total)^**a**^	Motivation to stay in the profession
Constant	5.30	3.01	4.73	3.40	3.51
Gender	0.01	−0.24	0.21*	− 0.13	0.22
Residence (vs. “> 100,000 inhabitants”)					
Rural (<=5000)	0.08	−0.49*	−0.23	− 0.05	0.50
Small and medium (<=100,000)	0.08	−0.20	−0.00	− 0.03	0.33
Children in the household (1 = yes)	−0.55***	−0.14	0.12	−0.30**	0.28
Work-Life Balance	−0.73***	−0.36***	0.13**	−0.44***	0.51***
R^2^	.48	.17	.08	.42	.23

*Note*: *p ≤ 0.05; +**p ≤ 0.01; ***p ≤ 0.001

^a^Computation of the total burnout score according to Kalimo et al. (2003)

## Discussion

Our results showed that there was a beneficial association between WLB and all dimensions of burnout as well as physicians` motivation to stay in the profession.

The average WLB in our sample was similar to the WLB found in highly demanding IT jobs and for teachers, and below the scores for managers in small and mid-sized companies [24, 25].

The fact that WLB was connected to burnout matches with results from other professions like banking and insurance [9, 10], and the fact that WLB was significantly associated with all three dimensions of burnout suggests that this connection is broad and also meaningful in practice. In addition, our results are in line with other studies that show connections between working overtime, long shifts, and burnout in medical professionals [26–28]. If future research strengthens a causal interpretation from WLB to burnout, WLB could be an avenue for improving GP mental health. Hence, measures that support GPs with administrative, legal and bureaucratic tasks as well as working fewer hours could help mitigate burnout risk. Beyond benefitting GPs and their patients, this could also help to reduce costs in the healthcare system that are caused by physician turnover and reduced working hours due to physician burnout [29].

Our results showed a positive connection between GPs` WLB and their motivation to stay in the profession, which matches with similar results in nurses and academics [30, 31]. In addition, research on health professionals connect better WLB to a reduced intention to leave the job [32, 33]. Studies also show that WLB is linked to a variety of other beneficial outcomes like affective commitment and job satisfaction [34, 35], and that organizations with a culture that values WLB could also benefit in terms of teamwork and safety [36]. Based on their analysis of the literature, Deery and Jago [37] suggest that WLB has become a key factor for employee management and retention, and a comparison of different generations across five countries implicates that the importance of WLB for attracting and retaining future talent may even increase due to changes in work-related values in younger individuals [38]. In that way, addressing GPs WLB is not only about GPs and patients, it is also a way to attract a new generation of talented physicians and to maintain and improve the healthcare system.

### Limitations

While our study has several advantages, e.g., being the first of its kind utilizing a sample of German GPs, it has also certain limitations. For example, we did not assess the number of years a physician spent at their workplace which may have an impact on WLB. In addition, future research may benefit from assessing interactions between WLB and burnout with a longitudinal design.

## Conclusions

The WLB of GPs is associated with burnout scores as well as with their motivation to stay in the profession. Therefore, interventions that improve WLB could be a way to not only improve GPs` mental health but also to strengthen and maintain the healthcare system and to attract a new generation of talented physicians.

## Data Availability

The dataset used and analyzed during the current study is available from the corresponding author on reasonable request.
